# Updates on Inflammatory Molecular Pathways Mediated by ADAM17 in Autoimmunity

**DOI:** 10.3390/cells13242092

**Published:** 2024-12-18

**Authors:** Margherita Sisto, Sabrina Lisi

**Affiliations:** Department of Translational Biomedicine and Neuroscience (DiBraiN), Section of Human Anatomy and Histology, University of Bari “Aldo Moro”, Piazza Giulio Cesare 1, I-70124 Bari, Italy; sabrina.lisi@uniba.it

**Keywords:** ADAM17, inflammation, autoimmunity

## Abstract

ADAM17 is a member of the disintegrin and metalloproteinase (ADAM) family of transmembrane proteases with immunoregulatory activity in multiple signaling pathways. The functional ADAM17 is involved in the shedding of the ectodomain characterizing many substrates belonging to growth factors, cytokines, receptors, and adhesion molecules. The ADAM17-dependent pathways are known to be crucial in tumor development and progression and in the modulation of many pathological and physiological processes. In the last decade, ADAM17 was considered the driver of several autoimmune pathologies, and numerous substrate-mediated signal transduction pathways were identified. However, the discoveries made to date have led researchers to try to clarify the multiple mechanisms in which ADAM17 is involved and to identify any molecular gaps between the different transductional cascades. In this review, we summarize the most recent updates on the multiple regulatory activities of ADAM17, focusing on reported data in the field of autoimmunity.

## 1. Introduction

Metalloproteinases (MMPs) are a set of enzymes that catalyze the proteolytic hydrolysis of peptide bonds, resulting in either the release of active molecules or the degradation of proteins. These proteolytic events are fundamental in all living cells to control processes such as tissue morphogenesis and remodeling, angiogenesis, inflammation, and autoimmunity, and unsurprisingly, they are also linked to pathological conditions when their functions are dysregulated [1]. In humans, there are numerous proteases identified in which essential roles in cellular physiology and biological processes are tightly regulated both transcriptionally and post-transcriptionally [2,3]. ADAM17 has been extensively studied in recent years, and multiple studies have focused on the molecular mechanisms of ADAM17 in diseased conditions. Over the last decade, ADAM17 has attracted considerable interest as an orchestrator of a wide range of autoimmune pathologies, largely demonstrated from research that employed genetic or inhibitory strategies in experimental mouse models [4,5,6,7]. This led researchers to believe that strategies based on blocking ADAM17 could have clinical implications. 

In this review, we provide an update on the complex biology of the ADAM17 protease and explore the current state of knowledge regarding the molecular and cellular regulatory mechanisms of ADAM17 in autoimmune conditions. Although many pieces of the puzzle have been identified, we still do not have an overview of the regulation of ADAM17 activity. This has prevented, to date, the initially promising use of strategies for the selective inhibition of ADAM17 for therapeutic purposes. However, we felt it necessary to insert a paragraph summarizing the experimental procedures implemented using ADAM17 inhibitor biomolecules. Despite their promise, the identified therapies were never used in the clinical setting due to multiple side effects.

## 2. Overview of ADAM 17’s Structure and Regulatory Mechanisms

**Figure 1:**
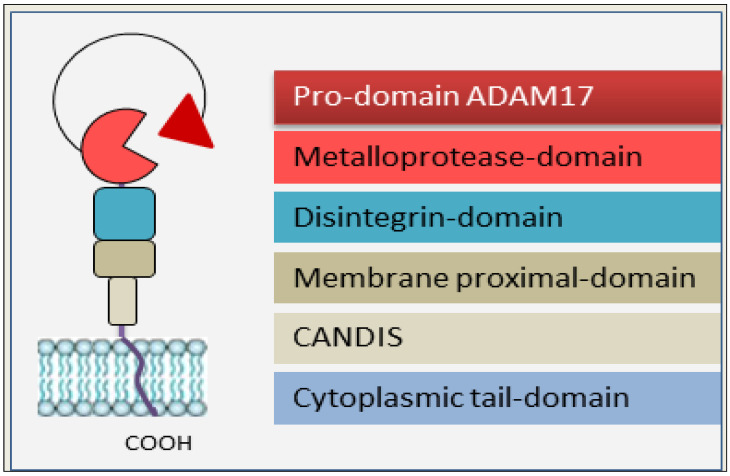
Schematic representation of ADAM17. ADAM17 is constituted of six different domains: a pro-domain, a metalloproteinase or catalytic domain, a disintegrin domain, a membrane-proximal domain, a conserved ADAM-seventeen dynamic interaction sequence (CANDIS), and terms with a cytosolic tail.

The protease ADAM17, also known as tumor necrosis factor (TNF)-α-converting enzyme (TACE), was discovered in 1988 [8]. ADAM17 was synthesized as a type I transmembrane cell-surface metalloprotease in inactive form. It is largely expressed in several tissues and cell types [9,10]. The ADAM17 protein is composed of a pro-domain, a metalloprotease domain, a disintegrin domain, a cysteine-rich membrane proximal domain, a CANDIS (conserved ADAM-seventeen dynamic interaction sequence) segment, and an intracellular cytoplasmic domain (Figure 1) [3]. A large amount of ADAM17 is stored within the endoplasmic reticulum in immature form, with the pro-domain still covalently attached. The maturation of ADAM17 in the Golgi apparatus occurs through the cleavage of the pro-domain by Furin, PC7, and PC5B pro-protein convertases [11,12]. Based on the process of synthesis and activation of ADAM17, it is possible to distinguish the catalytic form (80 kDa), the precursor form (110 kDa), and the glycosylated form (130 kDa) [13,14].

ADAM17 is widely regulated through several mechanisms, including proteolysis, phosphorylation, glycosylation, methylation, and acetylation [15]. Therefore, ADAM17 has numerous interactions with various proteins that modulate a myriad of intracellular signaling pathways. Recently, the membrane proteins iRhom1 and iRhom2 have been registered in the list of ADAM17 regulators and have become important components for the transport of ADAM17 to the cell surface and its activation [16,17]. They belong to the rhomboid protein family, which comprises the rhomboid intramembrane proteases iRhom1 and iRhom2. In several tissues, iRhoms appear to bind very specifically to ADAM17 and not to other ADAM proteins [17], aiding ADAM17 in surface transport via the cell membrane and thus facilitating the activation of ADAM17. These data are confirmed by the experimental use of iRhom-deficient mice in which ADAM17 maturation, trafficking, and proteolytic shedding of substrates were, as expected, abolished [16]. Furthermore, ADAM17 drives phosphorylation of the N-terminal cytoplasmic tail of iRhom2 through the involvement of mitogen-activated protein kinases (MAPKs). This phosphorylation process determines the detachment of ADAM17 from the complexes formed with iRhom2, thus promoting the proteolytic activity of ADAM17 [18].

ADAM17 participates actively, playing a central role in several epidermal growth factor receptor (EGFR) signaling pathways by shedding EGFR ligands as amphiregulin (AREG) or transforming growth factor (TGF-α) [19]. Indeed, it was demonstrated that ADAM17-mediated AREG shedding determines, subsequently, the activation of the ERK1/2 signaling pathway. The activation of the ADAM17/AREG/EGFR/ERK1/2 pathway leads to an increased release of pro-inflammatory cytokines in the salivary glands, augmenting the inflammatory status that characterizes the autoimmune rheumatic disease Sjögren’s syndrome (SS) [20]. Other investigators have also shown that phorbol-12-myristate-13-acetate (PMA) and EGF induce phosphorylation on ADAM17 in an ERK-dependent manner [4]. Given these different activation mechanisms, it is likely that ADAM17 activity is regulated in a tissue-specific and stimulus-specific manner [2,4,21]. A very recent discovery, deriving from the sad period of the severe acute respiratory syndrome coronavirus (SARS-CoV) pandemic, concerns the role played by virus cell entry. ADAM17 can cleave the SARS-CoV-1/2 receptor ACE2 and the SARS-CoV-2 spike protein, which is an important requisite for efficient infections [22].

Given the enormous number of discoveries that have been accumulating in recent years on the activation of ADAM17, numerous strategies have also been implemented to study the role of ADAM17 in the physiology and pathology of human tissues. Genetically manipulated homozygous Adam17ex/ex mice were created in the laboratory, characterized by eye, skin, and heart defects and reduced levels of soluble ADAM17 substrates [4,6]. These in vivo data confirm the observations made in several patients in which homozygous and heterozygous mutations in the ADAM17 gene have been associated with an inflammatory status in the skin, bowel, and heart [12].

As part of all these discoveries that improve knowledge of the mechanisms in which ADAM17 plays a fundamental role as an activator of traditional cascades, our interest lies in autoimmune diseases, which represent a thriving field of research, given the impact on the health of patients. ADAM17 has attracted considerable interest as a key enzyme in autoimmune disease pathogenesis since it is responsible for the release of soluble TNF-α. Increased TNF-α release is clearly correlated with dysregulated ADAM17 activity in autoimmune conditions [17,20,23], such as rheumatoid arthritis (RA), SS, systemic lupus erythematosus (SLE), psoriasis, and Crohn’s disease (CD) [17,24]. Furthermore, numerous recent discoveries support the correlation between autoimmunity, chronic inflammation, and organ fibrosis, which would occur through the activation of an epithelial–mesenchymal transition (EMT) program [11,25]. The following paragraphs will explore the most recent findings related to ADAM17-dependent pathways activated in autoimmune diseases.

## 3. Latest Discoveries on ADAM17-Mediated Pathways in Autoimmunity

Dysregulation of ADAM17-mediated signaling pathways is associated with several inflammatory autoimmune diseases [23], but the molecular mechanisms involved in this class of pathologies are not yet completely clear. In this paragraph, we will report the most recent discoveries on the involvement of ADAM17 in molecular features characterizing autoimmune diseases; this could help to guarantee the identification of common pathways based on which new therapeutic perspectives can germinate.

### 3.1. Updates on ADAM17-Mediated Interleukin (IL) Regulation (IL-6; IL-1; IL-15) in Autoimmune Diseases

#### 3.1.1. IL-6

Originally discovered as the shedding protein for the membrane-bound cytokine TNFα [8], in recent years, ADAM17 was identified to also be answerable for cleavage of the interleukin-6 receptor (IL-6R) [26] and approximately 80 additional transmembrane protein substrates [8].

**Figure 2:**
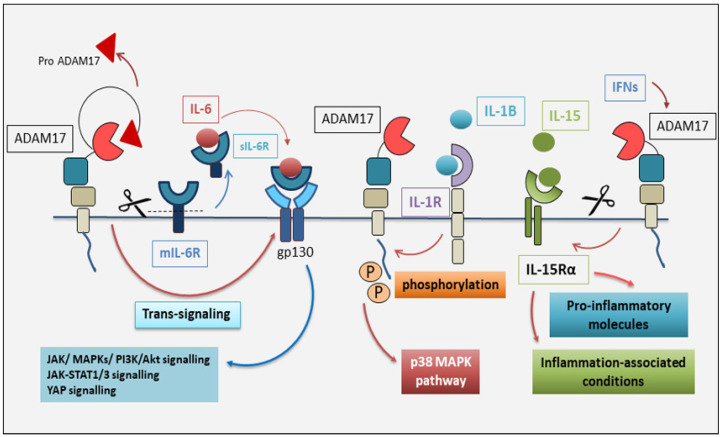
Interactions of ADAM17 with IL-6, IL-1β, and IL-15. ADAM17, through trans-signaling, cleaves the membrane interleukin-6 receptor (mIL-6R) to free a soluble molecule, which then associates with free IL-6 to constitute a signal-transducing receptor complex with gp130 and induce its dimerization. The dimerized gp130 leads to the activation of the JAK1/MAP kinase and PI3 kinase signaling and recruits the cytoplasmic transcription factors STAT1 and STAT3. Activation of gp130 also determines the phosphorylation and induction of the YAP pathway. The process of activation of ADAM17 is mediated by IL-1β that phosphorylates the cytoplasmic residue T735 and activates the p38 MAP kinase pathway. Interferons (IFNs) also upregulate cleavage of the cell surface IL-15Rα/IL-15 complex in an ADAM17-dependent manner, inducing the expression of inflammatory cytokines and subsequently an inflammatory status. ADAM17 (disintegrin and metalloprotease 17); gp130 (glycoprotein 130); IFNs (interferons); JAK1 (Janus kinase); MAPK (mitogen-activated protein kinase); IL-6 (interleukin 6); IL-1β (interleukin 1 beta); IL-15 (interleukin 15); STAT (signal transducer and activator of transcription); PI3K (phosphatidylinositol 3-kinase).

IL-6 is a four-helical immunoregulatory cytokine with pleiotropic actions, produced by several cell types upon opportune triggers and which can act on many cells during several disease conditions such as inflammatory states and the presence of tumors [27]. IL-6 performs its actions through linking to the IL-6R cleaved from ADAM17 [6]. On the target cells, the soluble IL-6R (sIL-6R) binds its ligand IL-6, and the complex of IL-6 and sIL-6R associates with gp130, activating homodimerization of signaling cascades. This method of signaling has been named IL-6 trans-signaling [28]. gp130 is a transmembrane protein expressed ubiquitously in all cells, while IL-6R is only present on some cells, such as hepatocytes, leukocytes, and epithelial cells. IL-6 is able to bind only to IL-6R but not to gp130, and thus IL-6 links only to cells that express IL-6R [26]. Since gp130 is expressed in all cells, IL-6 trans-signaling can lead to the induction of virtually every cell in the body [26]. The dimerized gp130 promotes induction of the tyrosine kinase Janus kinase 1 (JAK1), which is constitutively linked with the cytoplasmic domain of gp130, phosphorylating tyrosine residues within the cytoplasmic portion of gp130. This event triggers the engagement of the adapter protein and phosphatase SHP2 that activates MAP kinase and PI3 kinase signaling [29]. Furthermore, the phosphorylation of the tyrosine residues induces the recruitment of the transcription factors STAT1 and STAT3, which translocate into the nucleus, binding to DNA and promoting the transcription of gp130 target genes [29]. One of the former gp130 target genes is the gene coding for SOCS3. The SOCS3 protein is able to inhibit the activity of JAK1, and furthermore, it is a negative regulator of STAT3 activation [29]. Recently, it was demonstrated that gp130 induction leads to the phosphorylation and activation of the YAP pathway (Figure 2). This pathway was discovered to influence the development of autoinflammatory and autoimmune states [30]. This mechanism of the stimulation of the IL-6/sIL-6R complex leads to the activation of all gp130 proteins located on the cell surface, resulting in a higher signal spread [28].

#### 3.1.2. IL-1β

Different studies have demonstrated that the cytoplasmic domain of ADAM17 underwent phosphorylation when cells are treated with stimuli such as phorbol 12-myristate 13-acetate (PMA), EGF, various growth factors such as nerve growth factor (NGF) [21], fibroblast growth factor (FGF), transforming growth factor β (TGF-β), and interleukin-1β (IL-1β) [31].

In understanding the process of IL-1β-mediated activation of ADAM17, studies examining a threonine-to-alanine mutation at cytoplasmic residue 735 (T735>A) in ADAM17 have played a key role. These studies have highlighted how this mutation is sufficient to abrogate the ability of ADAM17 to respond and activate following stimulation with IL-1β, a process that involves the p38 MAPK pathway [31].

Phosphorylation of the cytoplasmic residue T735 of ADAM17 is, therefore, considered by some authors as a crucial step in the process of activation of ADAM17 mediated by IL-1β and the p38 MAP kinase pathway. The characteristic inhibitory function performed by T735 is given as an example, which must necessarily undergo phosphorylation to determine the activation of ADAM17. This activation would therefore be independent of the role played by its cytoplasmic domain [32]. On the other hand, some authors suggest that this mechanism can be considered feasible only in certain experimental conditions, while the stimulation of ADAM17 by IL-1β would depend exclusively on the activation of p38 MAPK, without involving the phosphorylation of T735 [32,33] (Figure 2).

#### 3.1.3. IL-15

IL-15 belongs to the four α-helix family of cytokines (including IL-2, IL-4, IL-7, IL-9, and IL-21) [34]. Differently from the receptors for IL-4, IL-7, IL-9, and IL-21, which are heterodimeric (a ligand-specific α chain binding the common γ (γc) chain), the receptors for IL-2 and IL-15 share an additional β chain (IL-2/15Rβ), leading to the formation of the heterotrimeric receptor for IL-15 [35]. Both IL-15Rα and IL-15 are expressed by a variety of tissues and cell types, including monocytes/macrophages, keratinocytes, fibroblasts, and nerve, muscle, and epithelial cells [36]. Alternative splicing of the IL-15Rα gene generates different isoforms of human and murine IL-15Rα [37]. Data relating to the activity of IL-15Rα were obtained using recombinant murine soluble IL-15Rα [38], which inhibits local inflammation induced by subcutaneous carrageenan administration in mice [39] and decreased cardiac allograft rejection in a mouse experimental model [40]. However, few data concerning the existence of natural sIL-15Rα are reported in the literature. Recently, the presence of the natural soluble IL-15Rα (sIL-15Rα) in mouse serum was demonstrated. Furthermore, murine fibroblasts constitutively release sIL-15Rα into the culture medium, and this process is further stimulated by PMA. Interestingly, both the constitutive and PMA-inducible IL-15Rα cleavages are ADAM17-dependent, as demonstrated in inhibitory studies [41]. A step forward has been made by demonstrating that type I interferons (IFNs) also upregulate the cleavage of cell surface IL-15Rα/IL-15 in an ADAM17-dependent manner [42]. This observation is the first demonstration that type I IFNs are a regulator of ADAM17 activity. Given the cleavage activities performed by ADAM17 on many pro-inflammatory molecules [23], it can be asserted that the proteolytic regulation of ADAM17 mediated by type I IFNs has the capability to alter immune responses during autoimmunity and inflammation-associated conditions (Figure 2).

### 3.2. ADAM17/Notch Signaling

It has been widely demonstrated, in recent years, that Notch activates an intracellular cascade, conserved throughout evolution, implicated in the regulation of various cellular processes [43]. For this reason, aberrant regulation of NOTCH or mutations in its coding gene may have pathological consequences [43].

In mammals, the Notch protein family includes four different Notch receptors (identified by Notch 1–4) [44]. Structurally, the four Notch receptor variants are type I membrane proteins, which present an extracellular ligand-binding domain (NECD) (N-terminal), a transmembrane domain (TMD), and an intracellular domain (NICD) (C-terminal) [44]. What characterizes each variant of the receptor is the NICD domain, necessary for receptor/ligand interactions and fundamental to then trigger the activation of the transcriptional cascade, which will lead to the modulation of the expression of specific target genes [45]. Notch is activated following binding to a specific ligand, which triggers a series of cascades of proteolytic cleavages. Ligands include five different single-pass transmembrane proteins belonging to the Serrate family (Jagged1 and Jagged2) and to the Delta-like family (delta-like 1 (DLL1) ligand, delta-like 3 (DLL3) ligand, and delta-like 4 (DLL4) ligand), which collectively belong to the Delta/Serrate/Lag-2 (DSLs) family [46]. Similar to the structure of Notch, DSL ligands are also transmembrane receptors, and for activation of NOTCH-mediated pathways to occur, a direct cell–cell interaction (trans-activation) must necessarily occur [46]. Following binding to Notch, NICD undergoes a process of ubiquitination via the E3 ligase Mind Bomb-1 [47]. This initiates a process of endocytosis of the Notch ligand/NECD complex within the cell, expressing the receptor acting as the ligand. It has been demonstrated that this endocytosis process is mediated by commonly involved factors such as clathrin, dynamin, epsin, and Picalm. Once this endocytosis process has started, the subsequent phases of NOTCH activation are characterized by successive proteolytic cleavages. Specifically, ADAM17 plays a primary role in the proteolytic cleavage of NOTCH, followed by the proteolytic activity of the gamma-secretase complex, which includes presenilin, PSENEN/PEN-2, APH1, and nicastrin [48]. Whether gamma-secretase cleavage occurs at the membrane or endosomal compartment is still under investigation. The result is the release of the intracellular active fragment, namely the NICD [49], into the cytosol and NICD translocation into the nucleus.

The nuclear translocation of NICD is followed by its binding to the DNA transcription factor RBP-Jκ (recombination signal binding protein for the immunoglobulin kappa J region) [49]. When NICD is not present in the nucleus, RBP-Jκ acts as a repressor of gene transcription, assisted in this activity by the co-repressor (Co-R) complex. The binding of NICD to RBP-Jκ, which follows its translocation into the nucleus, causes the conversion of RBP-Jκ to a transcriptional activator; this requires the recruitment of histone acetyltransferases (HAcs) and other coactivators called mastermind-like transcription factors (MAML) [50]. The union between NICD, RBP-Jκ, and MAML generates a Notch ternary transcriptional complex (NTC), which determines the cascade transcription of specific downstream genes, such as hairy/enhancer of split (Hes) and hairy/split enhancer with the motif YRPW (Hey) [51]. Hes and Hey proteins are proteins that act as repressors and have a key role in the regulation of various NOTCH-mediated phenomena, including the regulation of chronic inflammation that characterizes autoimmune diseases [52,53].

**Figure 3:**
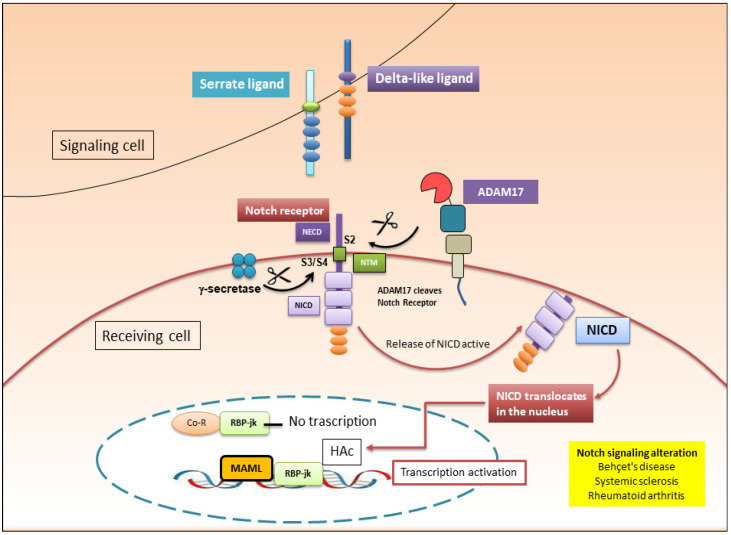
Notch signaling pathway. The Notch receptor binds to a Delta-like or Serrate family of ligands located on the cell. The Notch receptor is elaborated by ADAM17 at the S2 site in the NECD region and by γ-secretase at the S3/S4 site in the NTM region. The cleavage results in the release of the active component, NICD, in the cytoplasm. NICD enters the nucleus and associates with the conserved DNA-binding transcription factor RBP-Jκ. In the absence of interactions with the NICD, RBP-Jκ inhibits transcription by binding with the co-repressor (Co-R) complex. Otherwise, transcriptional induction results when the NICD links to RBP-Jκ in the nucleus, therefore converting RBP-Jκ from an inhibitor to an inductor of transcription, recruiting HAc and activating the MAML family, thus triggering the transcription of target genes. Co-repressor (Co-R); HAc (histone acetyltransferase); MAML (activation of mastermind-like); NECD (extracellular ligand-binding domain); NICD (Notch intracellular domain); Notch (neurogenic locus notch homolog protein); NTM (Notch transmembrane domain); RBP-Jκ (recombination signal binding protein for immunoglobulin kappa J region).

The activation scheme reported here represents the canonical NOTCH-mediated pathway (Figure 3); however, some authors have reported the existence of an alternative pathway of Notch activation that has the T cell receptor (TCR)/CD28 complex as its main actor, a process that appears to occur in the absence of ligands [54]. The study of this alternative route presents numerous doubts to be clarified.

#### Role of ADAM17/NOTCH Signaling in Autoimmune Diseases

The involvement of NOTCH in molecular mechanisms and complex cascade activations has been studied in various pathological conditions, but the correlation with the proteolytic activity of ADAM17 has not always been evaluated. In this section, we will analyze the most recent discoveries, analyzing the correlation between ADAM17 and NOTCH signaling in autoimmune diseases. An alteration of the complex mechanism of NOTCH activation has been demonstrated in various autoimmune diseases, and this represents a considerable step forward in the possibility of identifying therapeutic solutions that can reduce the state of chronic inflammation that characterizes these pathologies.

Behçet’s disease (BD) is an autoinflammatory disease characterized by altered transcription or mutations in several genes of currently unknown etiology, with ulcerative lesions affecting multiple organs [55,56]. Similarly to other autoimmune diseases, the characterization of the pathogenesis of BD is not yet complete; certainly, a genetic predisposition associated with environmental factors, lifestyle habits, and epigenetic modifications could predispose an individual to the onset of repeated inflammatory phenomena that tend to become chronic, as detected in BD pathology [56].

In reality, BD presents characteristics bordering on autoimmune diseases and auto-inflammatory conditions; like autoimmune diseases, it is closely related to alterations and mutations of the major histocompatibility complex (HLA), showing a close association with the HLA-B* allele 51 [57]; furthermore, it presents activated Th1 and Th17 cells and an overproduction of several pro-inflammatory cytokines [58]. Nonetheless, specific characteristics of autoinflammatory diseases, such as the presence of hyperactive neutrophils with the production of ROS (reactive oxygen species), phagocytosis, chemotaxis, and an absence of serum autoantibodies, have recently been highlighted in BD patients [59], with typical periods of remission and worsening of symptoms. In this intricate prospectus of potential mechanisms involved in the onset of BD, in recent years, an involvement of NOTCH signaling in BD was also evaluated and demonstrated. By analyzing the activation of the NOTCH-mediated transductional cascade in BD patients with uveitis, one of the hallmarks of BD [60], the authors demonstrated the ADAM-17/NOTCH-dependent activation of inflammatory mechanisms characterized by the consequent activation of Th17 [60]. This was confirmed by in vitro experiments based on the use of γ-secretase inhibitors, capable of preventing Notch activation by blocking its cleavage at the cell surface [60].

Systemic sclerosis (SSc) is considered an autoimmune disease with a high fibrotic component, which in recent years has been included among the diseases characterized by epithelial-to-mesenchymal transition (EMT)-dependent fibrosis [61,62]. The more advanced stages of the disease are characterized by an excessive accumulation of extracellular matrix (ECM) proteins. This appears to be determined by the activation of an EMT program, which leads to the differentiation of a very high number of fibroblasts [61,62]. This affects the functionality of various organs, modifying their architecture, and this represents the main cause of death in SSc patients [63]. The most studied pathogenetic mechanisms over the years have concerned the production of cytokines and pro-fibrotic factors such as TGFβ, PDGF, MCP-1, IL-4, and IL-13. Very recent research has highlighted a predominant role of transductional cascades mediated by the activation of Wnt, Hedgehog, and Notch in the pathogenesis of SSc [64]. Studies have been conducted on skin biopsy specimens collected from SSc patients and in vivo mouse models treated with ROS or bleomycin to induce an SSc-like syndrome. The unequivocal activation of ADAM17 has been demonstrated, which involves the proteolytic cleavage of NOTCH and the release of NICD in the cytoplasm with a consequent increase in the levels of the latter; furthermore, elevated levels of Jagged-1 and increased transcription of the target gene HES-1 were found in both the skin and lungs [64]. Confirmation of the involvement of the ADAM17/NOTCH pathway in SSc comes from studies that have used inhibitors, such as siRNAs directed against Notch genes, which prevented the development of fibrotic tissue in the mouse model [65]. The data obtained from similar studies have allowed us to hypothesize that the NOTCH transductional cascade acts in the phase of collagen synthesis in fibroblasts [66]. Therefore, any therapeutic treatments should act in the early stages of fibrotic evolution.

Rheumatoid arthritis (RA) is an autoimmune disease characterized by chronic inflammation of the joints that causes swelling, pain, and impaired function. Although multiple mechanisms have been studied as responsible for the pathogenesis of RA [67], and many have also been correlated with each other, the role of the ADAM17/NOTCH pathway has only recently been evaluated as a possible cause of the activation of genes expressed in an altered manner in RA [68]. Notch signaling appears to be activated in synovial cells, inducing these cells to produce an enormous amount of pro-inflammatory cytokines [68]. Furthermore, during experiments conducted on a murine macrophage model, overexpression of NICD was evaluated and demonstrated to be correlated with the activation of ADAM17, according to the canonical NOTCH signaling activation pathway. The increase in NICD expression was positively correlated with an increase in the production of TNF-α and interleukin (IL)-6 [69], once again supporting the involvement of ADAM17 as an activating factor of both cytokines evaluated.

Even in synovial cells similar to fibroblasts, activation of NOTCH has been demonstrated, leading to the exacerbation of the inflammatory condition, mediated by the production of TNF-α and IL-6 [70]. In support of this involvement of NOTCH in RA inflammatory conditions, it has also been demonstrated that in macrophages, IFN-γ, a powerful activator of these cells, determines the increase in the expression of jagged-1 with a simultaneous reduction in expression of the Delta-like family proteins DLL1 and DLL4 [71], resulting in a worsening of the clinical signs of RA. A confirmation of the canonical pathway of NOTCH activation in RA is derived from experimental mouse models in which rheumatoid arthritis is simulated with inclusion by collagen II. The use of a plasmid encoding Jag1 reduced the severity of the disease [72]. Similarly, the use of a neutralizing antibody against notch3 attenuated the inflammatory characteristics [73] (Figure 3).

### 3.3. Updates on the Involvement of the Axis ADAM17/Amphiregulin/EGFR in Autoimmune Diseases

AREG is a heparin-binding molecule capable of binding to the EGF receptor, EGFR [74]. The name amphiregulin (AREG) derives from its bifunctional activity. On the one hand, it is able to stimulate the growth of healthy or tumor cells; on the other, it can perform an inhibitory function on the proliferation of various tumor cell lines [75]. Synthesized from its gene, located in humans on chromosome 4q13-21, it initially takes the form of a transmembrane polarized glycoprotein (pro-AREG). This precursor, Pro-AREG, consists of an extracellular heparin-binding N-terminal domain, a hydrophobic transmembrane domain, and a carboxy-terminal domain with high homology to the corresponding EGF domain [76,77].

The AREG precursor is subsequently subjected to proteolytic cleavage, which leads to the release of the functionally active soluble form of AREG [75]. The proteolytic cleavage of the AREG precursor is carried out by ADAM17, and this is the reason why it is often referred to as the ADAM17/AREG axis [76,77].

After the binding of active AREG to EGFR, the consequence is the activation of intracellular cascade pathways that regulate cellular cycle and metabolism; furthermore, ADAM17/AREG-dependent pathways are involved in phenomena of chronic inflammation [78,79,80]. The activation of the ADAM17/AREG axis has been demonstrated in various inflammatory conditions and in various autoimmune diseases, characterized by a chronic inflammatory state [18,81,82].

Interesting was the demonstration that, during the activation or maintenance of an inflammatory state, AREG is expressed not only by cells of the immune system [77], but also by unexpected ones like the epithelial ones, now recognized as active players in the secretion or regulation of the levels of pro-inflammatory cytokines [81,83].

The mechanism underlying the interaction between AREG and EGFR has been clarified and is based on the need for EGFR to undergo dimerization and autophosphorylation in order to become active [84]. The dimerization of EGFR can also occur in the absence of a ligand, but it is now known that its activation depends on a conformational change following the interaction with the ligand, which brings two contiguous receptors closer together and induces their phosphorylation [85]. Unlike the EGFR ligands EGF or TGF-α, AREG has a lower affinity for EGFR due to a different amino acid positioned at the level of its receptor-binding domain [86]. This means that the receptor is not internalized and degraded at the lysosome level, resulting in constant activation of downstream pathways [86].

The ADAM17/AREG/EGFR axis determines the activation of various pathways, including Ras-Raf/MAPK, PI3K/Akt pathways, or pathways involving STAT activation, and lately attempts have been made to identify molecular bridges between all these mechanisms [87,88]. Current findings demonstrate that both the pro- and anti-inflammatory roles of AREG are implicated in the pathogenesis of autoimmune conditions. In psoriasis, overexpression of AREG in keratinocytes has been found, responsible for the inflammatory characterization of the disease [89]. Furthermore, abnormal expression of the ADAM17/AREG axis has been demonstrated in salivary gland epithelial cells (SGECs) in patients with Sjögren’s syndrome [81]. Along the same lines of research, EGFR blockade using tyrosine kinase inhibitors has proven to be a valid attempt to keep kidney damage under control in an experimental model of acute autoimmune glomerulonephritis (GN) [90]. In the same experimental setting, AREG gene silencing appears to have a significantly positive effect on renal protection from damage caused by altered inflammatory parameters [91].

The role of EGF and EGFR has been extensively studied in SS and correlated with the ADAM17/AREG activation axis [92,93]. All of these factors appear to be overexpressed in epithelial duct cells in the salivary glands, particularly in areas of lymphocyte infiltration and tissue alteration. The activation of the EGF/EGFR system in SS involves the metalloproteinase ADAM17 [20]. The identified mechanism demonstrated that, in SS, EGFR is a potent activator of the ERK1/ERK2 pathway (also known as the MAPK3/MAPK1 pathway). Elucidating signal transduction pathways, EGFR-mediated activation of ERK1/2 downstream effectors in SS SGECs depends on AREG activation in an ADAM17-dependent manner. This was corroborated by the use of AGREG-specific neutralizing antibodies, which significantly reduced EGFR transactivation and ERK1/2 phosphorylation. Furthermore, specific inhibitors of ADAM17 and EGFR led to a deactivation of the ADAM17/AREG/EGFR/ERK pathway and to a decrease in the synthesis of pro-inflammatory cytokines [20]. Other data in the literature indicate that an altered EGF/EGFR/ERK pathway is involved in the exacerbation of chronic inflammation in other autoimmune diseases, such as SLE [94] and psoriasis [95].

In contrast to these pro-inflammatory functions, AREG was also shown to have potent immunosuppressive effects. As a mechanism, AREG seems to act on Tregs to enhance their anti-inflammatory properties [96]. Independent research groups provided observations supporting an immunosuppressive role of AREG in experimental models of autoimmune uveoretinitis [97] and SLE [98].

Currently, researchers are aiming to prove that the ADAM17/AREG axis has an impacting role on the immune response, but sometimes contradictory, depending on whether the inflammation is acute, in which AREG would act in a pro-inflammatory manner, or chronic, in which AREG would predominantly have an anti-inflammatory effect.

**Figure 4:**
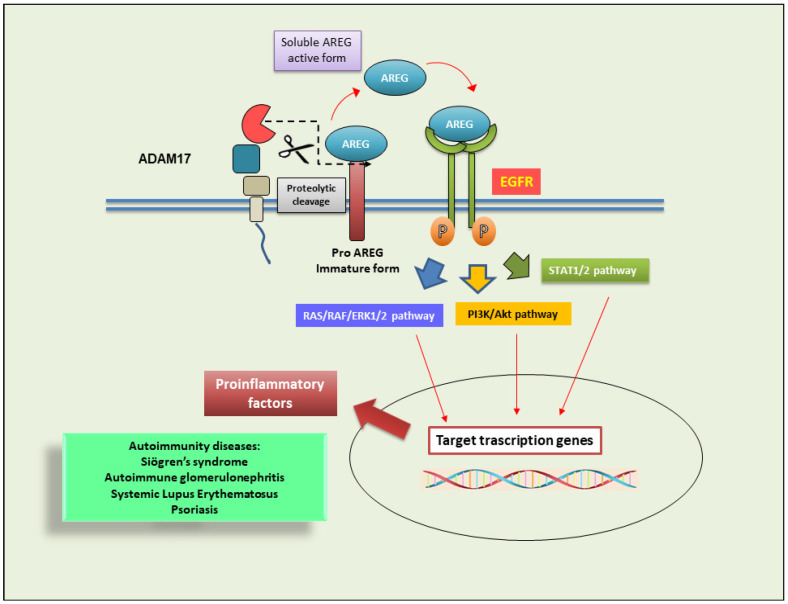
ADAM17 activation triggers AREG-dependent pathways. Mature ADAM17 cleaves EGFR which releases EGFR in mature form; mature EGFR binds soluble AREG and initiates EGFR-mediated intracellular signaling, including ERK 1/2, PI3K/AKT, and STAT1/2 pathways. These pathways are involved in chronic inflammation and autoimmune conditions. ADAM17 (disintegrin and metalloprotease 17); AKT (a serine/threonine protein kinase); amphiregulin (AREG); EGFR (epidermal growth factor receptor); pro-AREG (proamphiregulin); sAREG (soluble amphiregulin); ERK (extracellular signal-regulated kinase); PI3K (phosphatidylinositol 3-kinase); STAT1 (signal transducer and activator of transcription).

A schematic representation of the molecular pathways involving the ADAM17/AREG axis is reported in Figure 4.

### 3.4. ADAM17 Involvement in the EMT Program Activation in Autoimmune Diseases

In light of the latest discoveries on the roles played by ADAM17 in the pathogenetic mechanisms of autoimmune diseases, which we have discussed extensively in this review, an update on the role of ADAM17 in the phenomenon of epithelial-to-mesenchymal transition (EMT) could not be missed [99].

EMT represents a highly regulated cascade activation program characterized by the loss of the typical characteristics of epithelial cells that take on a mesenchymal appearance. The cells that undergo EMT, in fact, show an alteration and a decrease in epithelial adherens junctions, no longer present the apical–basal polarity, and show alterations at the cytoskeletal level [100,101]. By acquiring mesenchymal characteristics, these modified cells are largely responsible for the formation of metastases due to their high migration and replication capacity and are implicated in the fibrotic transformation of tissues, causing loss of functionality of the implicated organs [99,102].

**Figure 5:**
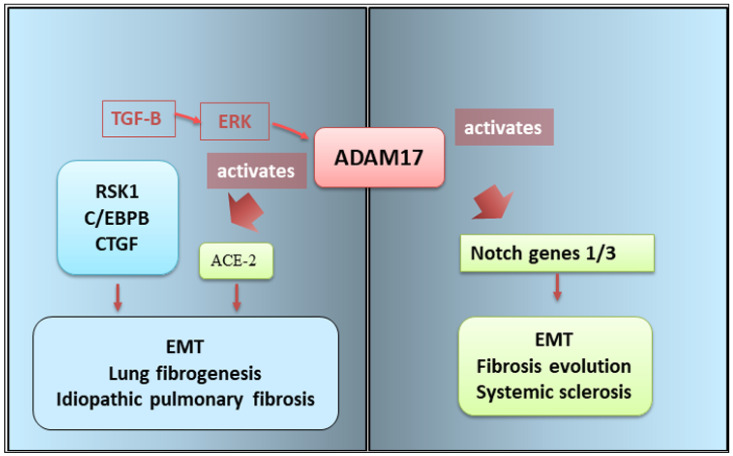
ADAM17 promotes fibrogenesis via EMT activation. The representative scheme shows the results of TGF-β-induced CTGF expression mediated via the ERK/ADAM17/RSK1/C/EBPβ pathway in lung epithelial cells, promoting the fibrosis process in idiopathic pulmonary fibrosis. ADAM17 activates Notch gene transcription that led to fibrotic conditions in systemic sclerosis. ADAM17 (a disintegrin and metalloprotease 17); C/EBPβ (CCAAT enhancer binding protein beta); CTGF (CCN2 cellular communication network factor); EMT (epithelial–mesenchymal transition); ERK (extracellular signal-regulated kinases); Notch (neurogenic locus notch homolog protein); RSK1 (ribosomal S6 kinase 1); TGF-β (transforming growth factor-beta).

Since persistent inflammation is certainly implicated in the fibrotic evolution of various autoimmune diseases, very recent research has evaluated the involvement of ADAM 17 in EMT-linked fibrosis [11] in these diseases. A correlation has been identified between the overexpression of ADAM17 and the degree of fibrotic evolution in patients with degenerative fibrotic evolution of autoimmune diseases. Furthermore, from a molecular point of view, ADAM17 represents a crucial factor in a series of transductional cascade mechanisms activated in EMT [103,104,105] (Figure 5).

Although the involvement of ADAM17 in the activation of the EMT program has been widely studied, the studies carried out concern the fields of oncology or fibrotic diseases. Attention is currently shifting to the field of autoimmune diseases, in which an EMT-dependent fibrotic evolution has been identified, and the results are very promising.

Idiopathic pulmonary fibrosis (IPF) is an autoimmune disease characterized by damage and activation of alveolar epithelial cells, infiltration of inflammatory cells, activation of an EMT program, and accumulation of extracellular matrix (ECM) proteins [104]. During the progression of IPF, lung epithelial cells undergo an EMT-mediated de-differentiation process, transforming into fibroblasts and causing an increasingly severe process of fibrotic degeneration with collagen deposition and architectural distortion [106]. Furthermore, a recent report demonstrated that ADAM17 determines the angiotensin-converting enzyme 2 (ACE-2) ectodomain shedding that was observed in lung fibrotic evolution in IPF [107].

In IPF, the ADAM17-mediated EMT activation is mediated by connective tissue growth factor (CTGF), a protein activated by TGF-β. CTGF was expressed at high levels in patients with IPF. The mechanism of activation seems to be triggered by TGF-β, which determines, in sequence, the activation of ERK, ADAM17, and ribosomal S6 kinase-1 (RSK1). The transduction cascade leads to the phosphorylation of enhancer-binding protein β (C/EBPβ), which activates CTGF gene transcription [108].

The same ERK/ADAM17/RSK1 pathway determines the phosphorylation and activation of C/EBPβ. Therefore, ADAM17 plays a key role in the induction of EMT in response to TGF-β, which occurs via the ERK/RSK1/C/EBPβ pathway [108].

Another autoimmune disease in which the role of ADAM17 in EMT-dependent fibrosis is recognized, albeit indirectly, is SSc, characterized by an evolution of fibrotic tissue in various organs affecting the instrumental, cardiovascular, urinary, and respiratory systems [61]. It has been shown that the EMT program is correlated with increased expression of Notch genes (1 and/or 3). Such increased expression has been described in various epithelial cells of SSc patients and appears to be correlated with the mesenchymal transformation of such cells [64]. This would be mediated by the activation of NOTCH genes by ADAM17, as described in the canonical activation pathway. An involvement of NOTCH genes in ADAM17-mediated EMT-dependent fibrosis was confirmed by experimental procedures of inhibition of the NOTCH-mediated signal transduction pathway, which led to a decrease or slowing down of the fibrotic process [64]. However, the actual molecular bridges that connect ADAM17 to EMT remain to be clarified, and, above all, it is of primary interest to evaluate these mechanisms in other autoimmune diseases. For an overview, see Figure 5.

## 4. ADAM17 as an Emerging Therapeutic Target for Autoimmune Diseases

Since ADAM17 has emerged as a protease that contributes to the pathogenesis of a multitude of disease states, a wide majority of small molecules synthesized so far as selective ADAM17 inhibitors were tested in tumors, chronic inflammatory diseases, and immune disorders [17,109]. More recently, investigations were warranted to assess the role of ADAM17 inhibitors in autoimmune diseases since elevated expression and/or activation levels of ADAM17 were observed in biopsies of patients affected by autoimmune diseases such as IPF, Crohn’s disease, RA, and psoriasis [2,17,110,111].

Unfortunately, a contributing factor to the lack of progress of ADAM17 inhibitors in the clinic has been the adverse side effects, such as hepatotoxicity, that accompanied the use of early-generation inhibitors of ADAM17 [109]. Indeed, many ADAM17-selective inhibitors used in experimental protocols have shown a lack of efficacy in Phase II clinical trials, as in RA patients’ treatment; currently, no molecule has passed the various evaluation phases in clinical trials, and, therefore, there are no drugs available on the market. In particular, ADAM17 inhibitors that initially seemed more promising, such as Apratastat (Wyeth Pharmaceuticals, Groton, Connecticut, USA), DPC 333 (Bristol-Myers Squibb Company, Princeton, NJ, USA), and INCB7839 (Incyte Corporation, Morges, Switzerland), showed toxic effects, and this led to the clinical trial being stopped [17,112]. Apratastat, in addition to the lack of effectiveness, was stopped for adverse events that occurred in RA patients. Similarly, DPC 333’s use in treating RA was ceased because it was ineffective and toxic to the liver and muscles [113].

By contrast, anti-TNF-α biological agents, such as etanercept and infliximab, had success as therapy in alleviating the clinical symptoms of RA and ameliorating patients’ lives. These antibodies were able to reduce the circulating level of TNF-α and act indirectly on ADAM17 inhibition. The most advanced among the biological drugs is INCB7839, a dual inhibitor of ADAM17 and ADAM10. Currently, it is used as therapy in association with rituximab for the treatment of non-Hodgkin lymphoma [111,114].

In addition, recently, an inhibitor of ADAM17 based on its pro-domain entered the clinic for inflammatory conditions such as inflammatory bowel disease (IBD) [111]. Interestingly, the recombinant pro-domain protein (A17pro) has been developed as an inhibitor of ADAM17 cell surface activity in vitro, while in vivo it was used in chronic autoimmune disease animal models [111].

In the last decade, researchers have identified more selective ADAM17-neutralizing monoclonal antibodies acting via different procedures, such as allosteric alteration of the binding capacity of the catalytic active site or inhibition of protein substrate linking [26]. These alternative modalities operate indirectly, targeting ADAM17 substrates (such as sIL-6R) or signaling effectors associated with ADAM17 activation. In this scenario, ADAM17 is considered an attractive therapeutic target since it could govern the IL6R trans-signaling pathway and, simultaneously, could block coexistent disease-associated ADAM17-regulated pathways [26]. For example, anti-IL-6R antibodies, named commercially as Tocilizumab, were approved for treatment of RA, and evidence has been collected for their beneficial effects on other systemic autoimmune diseases, including SLE, SSc, polymyositis, and large-vessel vasculitis [17]. On the basis of these considerations, molecules able to neutralize IL-6 trans-signaling in IPF were identified [115]. A recent study has demonstrated a temporal increase in ADAM17 in fibrotic lungs that mirrored increases in sIL-6Rα, supporting the role of ADAM17 in generating sIL-6Rα, largely from lung macrophages. In vivo neutralization of trans-signaling using the selective inhibitor recombinant gp130Fc resulted in a reduction in pulmonary inflammation and fibrosis associated with improvement in respiratory function. Thus, neutralization of trans-signaling attenuates disease and represents a promising new approach to treating IPF [115].

Within the scope of indirectly inhibiting the activation pathways mediated by ADAM17, very recently, data have been collected relating to the possibility of inhibiting Notch. The ubiquitous expression of Notch receptors and ligands on many cell types leads to the concrete possibility of identifying new therapeutic approaches in the field of autoimmunity [116,117].

Considering the high levels of ADAM17 found in psoriatic patients, this protease has become an important therapeutic target, having a crucial role in the pathogenesis of psoriasis. In a study conducted by Conway et al., GW3333, a dual inhibitor of matrix metalloproteinase (MMP) and ADAM17, was compared with other anti-TNF-α agents, demonstrating its effectiveness [116]. In addition, ADAM17 inhibitors were considered for the topical treatment of psoriasis because of their effect on T-cell-mediated immune response in psoriasis plaques [118].

Given the involvement of iRhom proteins in ADAM17-mediated mechanisms, emerging investigations have identified new inhibitors directed against the cytosolic tail of iRhom 1/2. However, it is necessary to carry out further studies to exclude that these inhibitors could, for example, block the interaction of iRhom2 with the stimulator of interferon genes (Sting), used in anti-inflammatory therapies [119]. Furthermore, after silencing iRhom 1/2 genes, an attenuation of excess TNF-α release in CD was observed, and the immunosuppressive capabilities of TGF-β signaling were restored, which ultimately reverses inflammatory tissue damage, suggesting a therapeutic option to treat inflammatory autoimmune diseases such as CD and RA [120].

Nowadays, interesting advancements in the field of synthetic short RNA molecules in preclinical studies suggest the repositioning of ADAM17 inhibitors, leading microRNAs (miRNAs) to be considered as a valid therapeutic alternative to classic drugs. A future goal could be to design miRNAs capable of interfering with the synthesis of pro-inflammatory cytokines. Overall, TNF-α, IL-1β, and IL-6 could be valid molecular targets in light of their deregulation in autoimmune diseases and their involvement as activators of transduction cascades involving ADAM17 [121,122,123,124].

Although the identification of therapeutically effective ADAM17 inhibitors is still complicated, and much has yet to be discovered and understood, it becomes more and more evident that there are many cogs in the ADAM17 regulatory machinery that may grant promising therapeutic strategies based on selective ADAM17 modulation.

## 5. Conclusions

In recent years, the dysregulated expression of ADAM17 was considered the driver of several pathological conditions, including autoimmune diseases. Despite the advances made in the knowledge of the basic mechanisms in which ADAM17 is involved in the processes of tissue homeostasis or development, the pathogenetic mechanisms in which it is implicated appear increasingly numerous and intricate. For this reason, the challenge is represented by the identification of molecular bridges that can connect these mechanisms together and thus have an overall vision. Obviously, it must be taken into account that the dysregulated expression and/or activation of ADAM17 depends on the substrate selectivity, the cellular context, and the specific stimulus that leads to the activation of this protease. Although the considerable amount of preclinical data give hope that regulation of ADAM17 represents a valid therapeutic possibility, due to ineffective or toxic implications, this has yet to translate into its broad clinical implementation. Furthermore, numerous scholars have used an indirect approach in the attempt to identify potential drugs represented by the blocking or regulation of ADAM17 substrates; attempts have been made to antagonize the activity of IL-6 or NOTCH, for example. But even in this case, harmful physiological effects have been highlighted, despite contrary observations coming from preclinical mouse models, which effectively tolerate the administration of IL-6 or NOTCH inhibitors or antibodies directed against ADAM17 and the pro-domain. In conclusion, in this review, we provide an overview of the most recent advances in the knowledge on the diverse molecular mechanisms involving ADAM17 activation in autoimmunity. The discussion of the mechanisms underlying ADAM17-mediated protein shedding could help scientists involved in translational studies to evaluate therapeutic prospects to counteract the chronic inflammation that characterizes these pathologies.

## Data Availability

Not applicable.
